# Supplementary material for: Content-rich biological network constructed by mining PubMed abstracts
Source: BMC Bioinformatics. 2004 Oct 8;5:147. doi: 10.1186/1471-2105-5-147 (PMC528731; doi:10.1186/1471-2105-5-147)
Supplement: Additional File 2 — The original results of the above study (non-essential files are deleted to keep the file size under the limit set by BMC bioinformatics). [file 1471-2105-5-147-S2.bz2 › chilibotAdditionalFile2/dip30/383ID10713102E9796/html/NTF3_NTRK3.html]

 


 **NTF3** and **NTRK3** 
  
Found 456 abstracts in PubMed, retrieved 30.  
 

 What does Google say? 
 PDF only 
| .edu only 

---

**Interactive relationship** (e.g. stimulation, inhibition, etc)

**Inhibitory relationship**- ... TrkC  [ **NTRK3** ]  receptors are responsible for neurotrophin 3  [ **NTF3** ]  inhibition.  Ref: 12424382 Science, 2002
- We also show that prolonged infusion of NT 3  [ **NTF3** ]  leads to a decrease in kindling induced Trk phosphorylation and a down regulation of the high affinity Trk receptors, TrkA and TrkC  [ **NTRK3** ] , suggesting an involvement of both cholinergic nerve growth factor receptors and hippocampal NT 3  [ **NTF3** ]  receptors in these effects.  Ref: 12453498 Neuroscience, 2002
**Neutral relationship**- Rat cerebral endothelial cells express trk C  [ **NTRK3** ]  and are regulated by neurotrophin 3  [ **NTF3** ] .  Ref: 12745089 Biochem Biophys Res Commun, 2003
- We also show that this processing, which does not occur in the TrkC  [ **NTRK3** ]  catalytic counterpart, is upregulated by NT 3  [ **NTF3** ]  and upon treatment with the tumor promoter 12 O tetradecanoylphorbol 13 acetate.  Ref: 12569366 Oncogene, 2003
- The embryonic Sertoli cells are the first testicular cells to differentiate and have been shown to express neurotropin 3 NT3  [ **NTF3** ] , which can act on high affinity trkC  [ **NTRK3** ]  receptors expressed on migrating mesonephros cells.  Ref: 12606390 Biol Reprod, 2003
- Here we show that p62 interacts not only with TrkA, but also with TrkB and TrkC  [ **NTRK3** ] , which are the primary receptors for brain derived neurotrophic factor and neurotrophin 3  [ **NTF3** ] .  Ref: 12471037 J Biol Chem< MedlineTA>J Biol Chem, 2003
- Nerve growth factor, brain derived neurotrophic factor and neurotrophin 3  [ **NTF3** ] , members of the neurotrophin family, bind to and activate TrkA, TrkB and TrkC  [ **NTRK3** ] , respectively, members of the Trk receptor tyrosine kinase family, to exert various effects including promotion of differentiation and survival, and regulation of synaptic plasticity in neuronal cells.  Ref: 12419535 Brain Res, 2002
- The distinct distribution of trkA receptors on small neurons and trkC  [ **NTRK3** ]  receptors on large neurons in the dorsal root ganglion correlates with the dependence of these two classes of neurons on nerve growth factor and neurotrophin 3  [ **NTF3** ] , respectively, for survival during development.  Ref: 12603263 Eur J Neurosci, 2003
- NT 3  [ **NTF3** ]  acts in an opposite fashion effecting a down regulation in BDNF expression in intact neurons and preventing reducing the injury associated increases in BDNF expression in both trkC  [ **NTRK3** ]  and nontrkC expressing subpopulations of injured neurons.  Ref: 12405958 Eur J Neurosci, 2002

**Non-interactive relationship** (e.g. studied together, co-existance, homology, etc.)

- Neurotrophin 3  [ **NTF3** ]  and its receptor TrkC  [ **NTRK3** ]  were not modified compared to controls.  Ref: 12677448 Acta Neuropathol , 2003
- Because neurotrophin 3  [ **NTF3** ]  NT 3  [ **NTF3** ]  is specifically produced by motoneurons and because interneurons express the NT 3  [ **NTF3** ]  high affinity receptor trkC  [ **NTRK3** ] , we examined the role of NT 3  [ **NTF3** ]  in the survival of PAX2 IR interneurons.  Ref: 12388583 J Neurosci, 2002
- In contrast, the expression levels of BDNF, NT 3  [ **NTF3** ] , trkB and trkC  [ **NTRK3** ]  were significantly higher at the rostral and caudal sites immediately adjacent to the maximal compression site.  Ref: 12774239 Acta Neuropathol Berl , 2003
- These cells express aberrant levels of functional p75 and TrkC  [ **NTRK3** ] , the putative high affinity receptor for the neurotrophin NT 3  [ **NTF3** ] .  Ref: 12616526 J Cell Biochem, 2003
- Cultured cortical progenitors express BDNF and NT 3  [ **NTF3** ] , as well as their receptors TrkB tyrosine kinase receptor B and TrkC  [ **NTRK3** ] .  Ref: 12832539 J Neurosci, 2003
- Consistent with a trkB.FL mediated mechanism, the i.t. administration of another trkB ligand, neurotrophin 4 5, also produces hyperalgesia while the trkC  [ **NTRK3** ]  agonist neurotrophin 3  [ **NTF3** ] , which weakly cross reacts with trkB, has little effect.  Ref: 12435470 Pain, 2002
- The role of the neurotrophin ligands, brain derived neurotrophic factor and neurotrophin 3  [ **NTF3** ] , and their respective tyrosine kinase receptors, TrkB and TrkC  [ **NTRK3** ] , in denervated taste buds is not known.  Ref: 12454994 J Comp Neurol, 2003
- Our results suggest that overexpression of BDNF, NT 3  [ **NTF3** ] , trkB and trkC  [ **NTRK3** ]  in motoneuron areas neighboring the site of mechanical compression may represent compensatory changes in response to the compromised neuronal function at the level of compression,.  Ref: 12774239 Acta Neuropathol Berl , 2003
- In single and double labeled immunofluorescent experiments, 09 taste bud sections from 13 hamsters were examined for immunopositive gemmal cells or nerve fibers using antibodies to BDNF and NT 3  [ **NTF3** ] , their respective receptors TrkB and TrkC  [ **NTRK3** ] , and the neural marker ubiquitin c terminal hydrolase L 1 protein gene product 9.5.  Ref: 12454993 J Comp Neurol, 2003
- The neurotrophins brain derived neurotrophic factor and neurotrophin 3  [ **NTF3** ] , as well as their respective tyrosine kinase receptors, TrkB and TrkC  [ **NTRK3** ] , influence peripheral target cell innervation, survival, and proliferation.  Ref: 12454993 J Comp Neurol, 2003
- In this work the expression and anatomical localization of NGF, BDNF and NT 3  [ **NTF3** ]  and of corresponding high affinity receptors TrkA, TrkB full length and truncated TR isoforms, TrkC  [ **NTRK3** ]  and of the low affinity p75 receptor were assessed in surgical samples from adult human lung by reverse transcriptase polymerase chain reaction RT PCR, Western blot and immunohistochemistry.  Ref: 12791675 Am J Respir Cell Mol Biol< MedlineTA>Am J Respir Cell Mol Biol, 2003
- METHODS Since the biologic function of NT 3  [ **NTF3** ]  is mediated primarily through Trk C  [ **NTRK3** ] , we investigated Trk C  [ **NTRK3** ]  immunoexpression on paraffin sections of 10 compound nevi and 63 melanomas.  Ref: 12753172 J Cutan Pathol, 2003
- Down regulation of NT 3  [ **NTF3** ]  and BDGF was the most significant, and TrkC  [ **NTRK3** ]  down regulation level was the lowest in receptors Trk family.  Ref: 12854135 World J Gastroenterol, 2003
- The TrkC  [ **NTRK3** ]  receptor ligand, NT 3  [ **NTF3** ] , did not promote inhibitory synapse development in the absence of neuronal activity, and such cultures exhibited hyperactive cortical discharges.  Ref: 12647813 Histol Histopathol, 2003
- Because TrkC  [ **NTRK3** ]  like IR in bud cells is absent, and sparse in fibers intragemmally and perigemmally, NT 3  [ **NTF3** ]  may also bind to bud cell TrkB so as to sustain fungiform gemmal cell viability post denervation.  Ref: 12454994 J Comp Neurol, 2003
- Mouse PTPRO mRNA was observed in neuron populations expressing TrkA, the high affinity nerve growth factor receptor, or TrkC  [ **NTRK3** ] , the neurotrophin 3  [ **NTF3** ]  receptor, and immunoreactive mPTPRO and TrkC  [ **NTRK3** ]  colocalized in large dorsal root ganglia proprioceptive neurons.  Ref: 12532410 J Comp Neurol, 2003
- Immunoreactivities for BDNF, NT 3  [ **NTF3** ] , trkB and trkC  [ **NTRK3** ]  were preferentially localized in the gray matter, particularly in the anterior horn cells.  Ref: 12774239 Acta Neuropathol Berl , 2003
- Western blot analysis confirmed these results, showing that the lacrimal gland expresses NGF, BDNF, NT3  [ **NTF3** ] , and NT4 as well as the NTrs TrkA, TrkB, and TrkC  [ **NTRK3** ]  and the p75 protein.  Ref: 12882781 Invest Ophthalmol Vis Sci, 2003
- To determine which of the neurotrophins NTs nerve growth factor NGF, brain derived neurotrophic factor BDNF, neurotrophin 3  [ **NTF3** ]  NT3  [ **NTF3** ] , and neurotrophin 4 5 NT4 and their receptors NTrs, TrkA, TrkB, TrkC  [ **NTRK3** ] , and p75, are present in the adult rat lacrimal gland.  Ref: 12882781 Invest Ophthalmol Vis Sci, 2003
- RT PCR showed positive bands at the appropriate sizes for NGF, BDNF, NT3  [ **NTF3** ] , and NT4, and for the receptors TrkA, TrkB, TrkC  [ **NTRK3** ] , and p75.  Ref: 12882781 Invest Ophthalmol Vis Sci, 2003
- In the current study, we tested whether submaximal concentrations of NT 3  [ **NTF3** ]  would maintain receptor tyrosine kinase TrkC  [ **NTRK3** ]  activation and Akt phosphorylation and thus promote long term survival of the pro OL against glutamate.  Ref: 12666655 Dev Neurosci, 2002
- However, despite overlapping expression of TrkB and TrkC  [ **NTRK3** ]  in many sensory ganglia, brain derived neurotrophic factor and neurotrophin 3  [ **NTF3** ]  null mutant mice display selective losses in neuronal subpopulations.  Ref: 12620975 Development< MedlineTA>Development, 2002
- For this purpose, serial immunohistochemical and immunoblot analyses were performed for the expression levels of endogenous brain derived neurotrophic factor BDNF, neurotrophin NT 3  [ **NTF3** ] , and their receptors, trkB and trkC  [ **NTRK3** ]  in 24 tip toe walking Yoshimura mice twy twy aged 12 24 weeks.  Ref: 12774239 Acta Neuropathol Berl , 2003
- We found that 1 trk C  [ **NTRK3** ] , a receptor for neurotrophin 3  [ **NTF3** ]  NT 3  [ **NTF3** ] , is dominantly expressed in RCEC, but trk B, a receptor for brain derived neurotrophic factor, is dominantly expressed in RAEC.  Ref: 12745089 Biochem Biophys Res Commun, 2003
- This study shows that, in addition to alternative splicing, ectodomain shedding represents a novel means of regulating TrkC  [ **NTRK3** ]  receptor signaling, and consequently NT 3  [ **NTF3** ]  biological effects on target cells.  Ref: 12569366 Oncogene, 2003
- Our results demonstrate that NT 3  [ **NTF3** ]  at any concentration sufficient to activate the TrkC  [ **NTRK3** ]  receptor results in a transient phosphorylation of the receptor and of Akt due, in part, to downregulation of the Trk receptor.  Ref: 12666655 Dev Neurosci, 2002
- the numbers of neurons expressing the NT 3  [ **NTF3** ]  high affinity receptor trk C  [ **NTRK3** ]  were unchanged.  Ref: 12598727 Neuroreport, 2003
